# Deciphering cytopenias in internal medicine: a single-center observational study

**DOI:** 10.1007/s11739-023-03517-z

**Published:** 2024-01-25

**Authors:** Simona Leoni, Marta Ferraresi, Irene Motta, Cinzia Hu, Anna Ludovica Fracanzani, Wilma Barcellini, Bruno Fattizzo

**Affiliations:** 1 Fondazione IRCCS Ca’ Granda Ospedale Maggiore Policlinico, Via F. Sforza 35, 20100 Milan, Italy; 2 University of Milan, Milan, Italy; 3 Department of Clinical Sciences and Community Health, University of Milan, Milan, Italy; 4 Department of Pathophysiology and Transplantation, University of Milan, Milan, Italy; 5 Department of Oncology and Hemato-Oncology, University of Milan, Milan, Italy

**Keywords:** Anemia, Thrombocytopenia, Neutropenia, Bone-marrow evaluation, Transfusions, Internal medicine

## Abstract

Cytopenia is a common finding in patients admitted to internal medicine wards and the clinical workup may be long and time-consuming. In this single-center observational study, we analyzed a series of 151 inpatients who received hematologist referral due to cytopenia observed during hospital admission. Patients were mainly elderly (median 71 years, 15–96) and 87% had at least one comorbidity. Anemia was the most common cytopenia (91%), followed by thrombocytopenia (51%), and neutropenia (22%); 73 (48%) patients had a bicytopenia and 5 (3%) pancytopenia. Cytopenias were mainly severe, 66% of cases required RBC transfusions, and 21% platelet pools. During a median hospital stay of 15 days (1–166), 53 subjects (35%) received a hematologic discharge diagnosis, whilst the two-thirds had secondary cytopenia mainly due to associated comorbidities. Only about 34% of 2,728 diagnostic tests performed (including laboratory, imaging, and histology) clearly informed the discharge diagnosis in this heterogenous setting. Specifically, bone-marrow evaluation indicated in 46 (30%) patients, was diagnostic in 32 (69.6%). Eleven percent of patients died due to progression of the oncohematologic disease (29%), sepsis (24%), and solid tumor progression (24%). In conclusion, cytopenias in the internal medicine setting are mainly severe, more frequently secondary to associated comorbidities (2/3 of patients) and deserve proper workup before second/third-level tests (immune-hematological assays and CT scan or PET and bone-marrow evaluation, respectively).

**Supplementary Information:**

The online version contains supplementary material available at 10.1007/s11739-023-03517-z.

## Introduction

Cytopenia represents a common clinical finding in patients admitted to internal medicine and surgical wards [1–3]. The underlying causes are often primarily non-hematological and include nutrients deficiencies, chronic diseases, inflammatory state, and cancer [4, 5]. The differential diagnosis may be challenging, since most patients are elderly and carry several comorbidities that may further confound the picture [6]. Hematologist consultation may lead to a number of specialistic laboratory and instrumental investigations. The utility and reliability of the latter during admission may be questionable, particularly due to concomitant acute conditions, such infections, and drug use. In addition, the lack of predictors of hematologic diagnoses in these patients does not aid to discriminate those needing second-level tests, such as bone-marrow (BM) aspirate and trephine. Whilst computer tomography (CT) and positron emission tomography (PET) scans are largely and autonomously used by internal medicine specialists, BM evaluation is generally indicated by the hematologist and its interpretation carries several drawbacks [7]. In this study, we analyzed a series of inpatients who received hematologist referral due to cytopenia observed during hospital admission. We focused on the laboratory and radiological investigations performed since admission until diagnosis, including those asked by the hematologist, to identify possible indicators of underlying hematologic condition. Furthermore, we analyzed mortality rate and causes among cytopenic patients and evaluated possible clinical and laboratory associations.

## Methods

### Design of the study

This is a retrospective cohort study conducted between January 2018 and December 2019 at IRCCS Fondazione Ca’ Granda—Ospedale Maggiore Policlinico (Milan, Italy). The study was approved by the local Ethical Committee as a sub-study of the CYTOPAN observational trial [NCT05931718] and was conducted according to the Helsinki Declaration.

Patients admitted to non-hematological wards who were referred to the hematologist due to anemia (Hb < 12 g/dl), thrombocytopenia (platelets, PLT < 100.000/mmc) or neutropenia (absolute neutrophil count, ANC < 1500/mmc) without a known hematological diagnosis were included. Patients were stratified according to age (< 65 years, between 65 and 79, and ≥ 80) and according to the severity of the cytopenia [8, 9].

### Clinical and hematologic workup

Hematologic parameters, including hemolytic markers (LDH, haptoglobin, total and fractionated bilirubin, and absolute reticulocytes), immune-hematologic evaluation (anti-PLT autoantibodies, direct and indirect antiglobulin test, DAT and IAT, ANA, ENA, anti-DNA, and anti-phospholipid antibodies), serology for major hepatotropic viruses (HBV, HCV), HIV, Herpes viruses (CMV, EBV) and Parvovirus B19, as well as the isolation of bacterial or fungi on cultural exams, were recorded. Furthermore, BM evaluation (aspirate and trephine) and imaging studies (ultrasound sonography, US, CT, PET) reports performed from initial admission to cytopenia diagnosis were registered. Presence of comorbidities was also collected. The following comorbidities were considered: cardiovascular disease (congestive heart failure, arrhythmias, ischemic cardiomyopathy, other cardiomyopathies, arterial hypertension, peripheral vasculopathy, valvulopathy, presence of intravascular devices), kidney disease, hepatic disease (acute hepatitis, chronic hepatopathy, cirrhosis, hepatocellular carcinoma), autoimmune disease, diabetes mellitus, thyroid disease, sepsis, and thrombosis.

### Treatment and outcome

All therapies administered during admission were recorded with particular attention to those indicated for the cytopenia [10–12]. The final diagnosis was registered as reported by the discharging physician and classified as hematologic or non-hematologic, and the former distinguished in oncologic and non-oncologic. Hematologic diagnoses were then classified according to current WHO classification of myeloid and lymphoid diseases [13]. A comparison of baseline demographic, clinical and laboratory features, including cytopenia type and severity and presence of comorbidities, was performed between patients receiving or not a hematological diagnosis.

Occurrence of death and its cause were also collected. Mortality analysis included the evaluation of clinical and laboratory variables (age and gender, diagnosis, hematologic parameters, comorbidities, and therapies) possibly associated with fatal outcome.

### Statistical analysis

Descriptive statistics was applied to describe the distribution of variables among the different subgroups of patients. Student *t* test was used to compare continuous variables, Chi-square and Fisher exact test were applied to evaluate categorical ones. The sensitivity, specificity and positive predictive value for DAT and anti-PLT antibodies was also calculated.

## Results

### Demographics

A total of 170 hematologic consultations were evaluated: 151 were included since had been performed for new onset cytopenia, whilst 19 were excluded since regarded patients already on hematologic follow-up (Supplementary Fig. 1). Patients were mainly elderly (median age 71 years, range 15–96; 33% of patients > 80 and 32% between 65 and 80 years), and male to female ratio was 1.25 (Table 1). Regarding medical history, 87% of patients had at least one comorbidity, mainly cardiologic (59%) and liver diseases (39%), followed by kidney disease, diabetes, and autoimmune diseases (about 20% each) (Supplementary Table 1).

**Table 1:** Demographics and reason for admission

	All *N* = 151	Non-hematologic *N* = 98	Hematologic *N* = 53
Age, years (range)	71 (15–96)	68 (15–96)	79 (19–93)*
Males	84 (55.6%)	62 (63.3%)	22 (41.5%)
Females	67 (44.6%)	36 (36.7%)	31 (58.5%)*
Reason for admission			
Cytopenia	53 (35.1%)	20 (20.4%)	33 (62.3%)
Fever	27 (17.9%)	20 (20.4%)	7 (13.2%)
Cardiovascular	8 (5.3%)	8 (8.2%)	0
Acute/chronic kidney injury	12 (7.9%)	9 (9.2%	3 (5.7%)
Acute/chronic liver disease	18 (11.9%)	15 (15.3%)	3 (5.7%)
Bleeding	13 (8.6%)	11 (11.2%)	2 (3.8%)
Acute respiratory failure	11 (7.3%)	8 (8.2%)	3 (5.7%)
Other	41 (27.2%)	30 (30.6%)	11 (20.8%)
Admission in clinical ward	134 (88.7%)	81 (82.7%)	53 (100%)
Admission in surgical ward	17 (11.3%)	17 (17.3%)	0
Median hospital stay, days	15 (1–166)	15 (1–166)	15 (4–47)
Blood counts			
Hb (g/dL)	8.4 (3.3–16.5)	8.6 (3.3–15.6)	8.0 (3.5–16.5)
PLTs (10^3/mmc)	99 (2–754)	111 (93–754)	87 (2–384)
ANC (10^3/mmc)	3.1 (0.02–29.86)	3.22 (0.02–29.86)	2.82 (0.04–11.3)

*p < 0.05

*Hb* hemoglobin, *PLTs* platelets, *ANC* absolute neutrophil count; values are expressed as N(%) unless otherwise specified

Most patients were admitted to a clinical ward (89%), whilst a minority (11%) in a surgical one. The main reason for admission was peripheral cytopenia (35%), followed by liver/kidney diseases (25%), fever (18%), and respiratory/cardiologic events (12%). A minority of patients had been admitted due to acute or chronic bleeding (9%).

### Distribution of cytopenias and discharge diagnoses

The most common cytopenia was anemia (91% of cases), followed by thrombocytopenia (51%), and neutropenia (22%). Seventy-three (48%) patients had a bicytopenia and 5 (3%) pancytopenia.

Median hospital stay was 15 days (1–166) and only 35% of subjects received a hematologic discharge diagnosis (Table 2), whilst the two-thirds had secondary cytopenia mainly due to associated comorbidities. Hematologic diagnoses were equally distributed between oncological (51%) and benign forms (49%). Specifically, the most frequent oncohematologic diagnoses were myelodysplastic syndrome (MDS, 29%) and acute myeloid leukemia (30%), followed by non-Hodgkin lymphoma (NHL, 15%) and multiple myeloma (4%). Non oncohematological diagnoses included autoimmune hemolytic anemia (AIHA) and immune thrombocytopenia (ITP) (27% each). Patients receiving a hematological diagnosis were significantly older (median 79 vs 68 years, *p* = 0.02) and more frequently female (59% vs 36%, *p* = 0.01). Regarding cytopenia, an association with hematological discharge diagnosis was noted with thrombocytopenia (64% vs 44%, *p* = 0.02) and neutropenia (28% vs 19%, *p* = 0.008), but not with anemia; the severity of the cytopenias and the presence of bicytopenia or pancytopenia were not significantly associated with a hematologic discharge diagnosis. Hematologic patients more frequently displayed cardiologic comorbidities (72% vs 52%, *p* = 0.01), mainly arterial hypertension (84%), followed by atrial fibrillation (37%). All patients admitted to surgical wards had a non-hematological final diagnosis. Seven patients were discharged without a definitive hematological diagnosis that was, however, confirmed during the follow-up (4 MDS, and 3 indolent NHL).

**Table 2:** Discharge diagnosis and outcome

	All	Anemia	Thrombocytopenia	Neutropenia
	*N* = 151	*N* = 137	*N* = 78	*N* = 34
Hematologic diagnosis	53 (35.1%)	47 (30.7%)	34 (43.6%)*	15 (44.1%)*
Non-oncohematologic	26 (16.6%)	22 (46.8%)	17 (50%)	3 (20%)
Autoimmune hemolytic anemia	7 (26.9%)	7 (31.8%)	1 (5.9%)	1 (33.3%)
Immune thrombocytopenia	7 (26.9%)	5 (22.7%)	7 (41.2%)	0
Multifactorial anemia	4 (15.4%)	3 (13.6%)	4 (23.5%)	1 (33.3%)
Vitamin or iron deficiency anemia	3 (11.5%)	3 (13.6%)	1 (5.9%)	0
Chronic disseminated intravascular coagulation	1 (3.8%)	1 (5.5%)	1 (5.9%)	0
Thrombotic microngiopathy	1 (3.8%)	0	1 (5.9%)	0
Iatrogenic	2 (7.7%)	2 (9.1%)	1 (5.9%)	1 (33.3%)
Other	1 (3.8%)	1 (5.5%)	1 (5.9%)	0
Oncohematologic	27 (18.5%)	25 (53.2%)	17 (50%)	12 (80%)
Myelodisplastic syndrome	8 (29.7%)	7 (28%)	6 (35.3%)	3 (25%)
Diffuse large B-cell lymphoma	1 (3.7%)	1 (4%)	1 (5.9%)	0
Large granular lymphocytic leukemia	1 (3.7%)	1 (4%)	0	1 (8.3%)
Acute myeloid leukemia	5 (18.5%)	5 (20%)	4 (23.5%)	5 (41.7%)
Non-hodgkin lymphoma	2 (7.4%)	1 (4%)	1 (5.9%)	0
Acute myelo-monocytic leukemia	2 (7.4%)	2 (8%)	1 (5.9%)	2 (16.7%)
Multiple myeloma	1 (3.7%)	1 (4%)	0	0
Diagnostic definition in progress	7 (25.9%)	7 (28%)	4 (23.5%)	1 (8.3%)
Infection	53 (35.1%)	48 (35%)	27 (34.6%)	14 (41.2%)
Cardiovascular	15 (9.9%)	15 (10.9%)	9 (11.5%)	0
Solid tumor	23 (15.2%)	21 (15.3%)	14 (17.9%)	4 (11.8%)
Autoimmune disease	15 (9.9%)	15 (10.9%)	6 (7.7%)	4 (11.8%)
Death	17 (11.3%)	16 (11.7%)	12 (15.4%)	5 (14.7%)
Put on follow-up	62 (41.1%)	55 (40.1%)	34 (43.6%)	13 (38.2%)

*p < 0.05; values are expressed as N(%)

Regarding comorbidities, anemia was significantly associated with chronic kidney disease (26% vs 0%, *p* = 0.04), neutropenia with autoimmune disease (35% vs 15%, *p* = 0.01), and thrombocytopenia with liver disease (55% vs 27%, *p* = 0.09), although not significantly (Table 2).

### Diagnostic tests

A total of 2,728 diagnostic tests were performed, resulting in about 18 tests per patient, including laboratory and imaging investigations, and bone-marrow evaluations. Overall, only about 34% of tests reported a result clearly informing the discharge diagnosis. Table 3 shows the most relevant tests. Regarding immune-hematological investigations, 6 patients (12%) among those tested showed positive DAT and 6 (12%) positive IAT. All DAT positive patients also presented altered hemolytic markers and received a discharge diagnosis of AIHA. This resulted in a sensitivity of 100% and a specificity of 97%. Anti-PLTs antibodies were detectable in 27 patients (56% of tested cases), of whom only 7 received a final diagnosis of ITP. The calculated sensitivity and specificity were 86% and 49%, respectively. Anti-neutrophil antibodies were positive in only 1 patient (25% of tested cases). In addition, 17 patients (34% of those tested) showed ANA positivity, 4 (10%) positive ENA antibodies, and 2 (8%) anti-phospholipid antibodies.

**Table 3:** Diagnostic tests

	All *N* = 151	Non-hematologic *N* = 98	Hematologic *N* = 53
Blood counts (*N* = 151)			
Hb (g/dL)	8.4 (3.3–16.5)	8.6 (3.3–15.6)	8.0 (3.5–16.5)
PLTs (10^3/mmc)	99 (2–754)	111 (93–754)	87 (2–384)
ANC (10^3/mmc)	3.1 (0.02–29.8)	3.2 (0.02–29.8)	2.8 (0.04–11.3)
Other blood tests			
Iron (mcg/dL) (*N* = 135)	68 (13–284)	64 (13–284)	79 (14–282)
Ferritin (mcg/L) (*N* = 136)	587 (3–15.266)	537 (3–15.266)	572 (13–4.094)
Transferrin (mg/dL) (*N* = 123)	166 (59–399)	159 (59–399)	178 (97–345)
Vitamin B12 (ng/L) (*N* = 127)	444 (100–1.798)	461 (100–1.798)	420 (151–1.394)
Folate (*N* = 126)	5 (0.1–602)	4.8 (0.08–19.2)	5.2 (0.9–602)
Immunohematologic parameters			
DAT (*N* = 52) positive	6 (11.5%)	0	6 (33.3%)
IAT (*N* = 50) positive	6 (12%)	3 (8.8%)	3 (18.8%)
Anti-PLT (*N* = 48) positive	27 (56.3%)	13 (48.1%)	14 (66.7%)
Anti-neutrophil (*N* = 4) positive	1 (25%)	0	1 (33.3%)
Autoantibodies			
ANA (*N* = 50) positive	17 (34%)	7 (26.9%)	10 (41.7%)
ENA (*N* = 39) positive	4 (10.3%)	3 (15%)	1 (5.3%)
Anti-DNA (*N* = 36) positive	2 (5.6%)	2 (11.8%)	0
LLAC (*N* = 24) positive	1 (4.2%)	1 (11.1%)	0
Serology			
HIV (*N* = 64) positive	1 (1.6%)	1 (2.4%)	0
HBV (*N* = 96) positive	8 (8.3%)	5 (8.5%)	3 (8.1%)
HCV (*N* = 92) positive	5 (5.4%)	3 (5.4%)	2 (5.5%)
CMV (*N* = 69) positive	11 (15.9%)	8 (19%)	3 (11.1%)
EBV (*N* = 65) positive	4 (6.1%)	3 (7.3%)	1 (4.2%)
Parvovirus (*N* = 56) positive	2 (3.6%)	1 (3.1%)	1 (4.2%)
Imaging tests			
Ultrasonography (*N* = 151) positive	46 (30.5%)	29 (29.6%)	17 (32.1%)
Computed tomography (*N* = 71) positive	19 (26.8%)	9 (17.6%)	10 (50%)*
Positron emission tomography (*N* = 20) positive	12 (7.9%)	6 (46.1%)	6 (85.7%)
Bone-marrow biopsy (*N* = 46)			
Diagnostic alterations	40 (86.9%)	11 (79%)	29 (90%)

*p < 0.05

*Hb* hemoglobin, *PLTs* platelets, *ANC* absolute neutrophil count, *DAT* direct antiglobulin test, *IAT* indirect antiglobulin test, *ANA* anti-nuclear antibodies, *ENA* extractable nuclear antigen antibodies, *LLAC* lupus-like anticoagulant

Concerning imaging, splenomegaly was found at US in 46 (31%) patients, 17 (32%) of whom received a oncohematologic diagnosis at discharge. Contrast enhanced whole body CT scan was performed in 71 (37%) patients and infections were the most frequent finding (28%), followed by splenomegaly (23%). Only 20 (13%) patients underwent PET scan: lymph node hypercaptation was the most common result, which was equally distributed between hematologic and non-hematologic patients. Spleen and bone-marrow hypercaptation were also frequent (35% and 30%, respectively). The finding of lymphoadenopathy and splenomegaly at CT scan was significantly more frequent in patients receiving a hematologic diagnosis (50% vs 17.6%, *p* = 0.04). Similarly, there was a trend for higher spleen and bone hypercaptation at PET examination in this group.

Bone-marrow evaluation was indicated by the hematologist in 46 (30%) patients, 32 (69.6%) of whom received a hematological diagnosis. Median cellularity at bone-marrow trephine was 50% (10–95%). Dyserythropoiesis was present at most examinations (80%), whilst dysmegakaryopoiesis, dysgranulopoiesis and fibrosis were reported in a minority of cases. Finally, in most trephines (57%) a mixed B-/T-cell lymphoid cellular infiltrate was detected, and 4 patients with clonal lymphoid infiltrate received a final diagnosis of NHL (Supplementary Table 2).

**Fig. 1:**
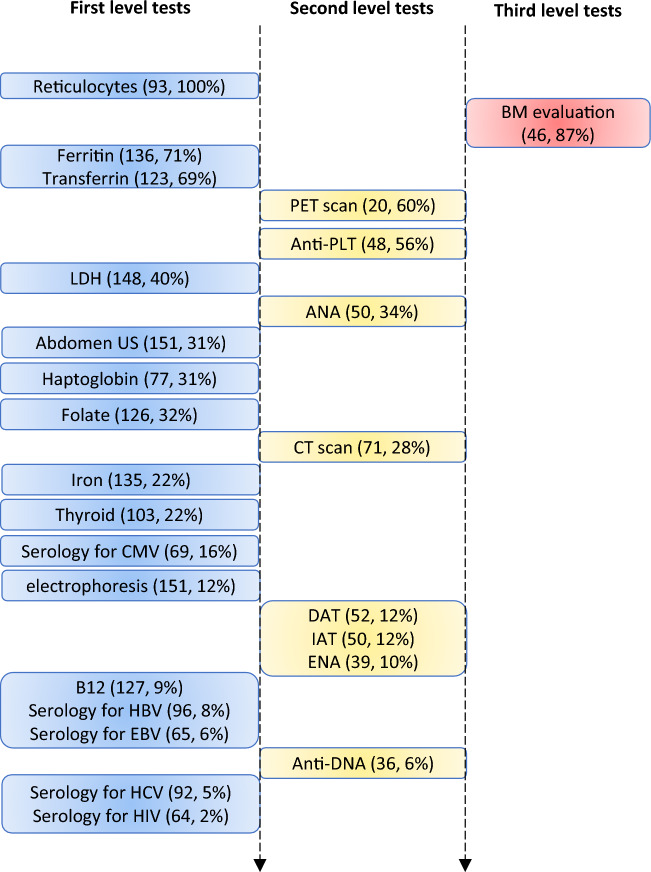
Different diagnostic tests for cytopenias according to percentage of alteration and level of investigation. Number and percentage of alteration or positivity of the test are reported in brackets

Figure 1 displays the different tests performed according to the frequency of alteration and the level of investigation: first level (blood counts, nutrients, serology for common viral infections, and abdomen ultrasound), second level (immune-hematology tests, organ and non-organ specific autoantibodies, CT and PET scan), and third level (bone-marrow evaluation).

### Therapies administered for cytopenia

During admission, 99 patients (66%) required RBC transfusions, 31 (21%) platelet pools, whilst only 12 (8%) needed fresh plasma concentrates; 56 subjects (37%) required folic acid supplementation, 6 vitamin B12 (10% both), and 11 (11.7%) received iv iron infusion. Steroid therapy was initiated in 59 (39%) patients. Other treatments included antibiotics (66%), neuroleptics (13%), and substitutive levothyroxine (14%).

### Outcome

A total of 17 patients (11.3%), 6 of whom with a confirmed hematological diagnosis, died during hospitalization (Supplementary Table 3). Median age was 70 years (19–94), and median duration of hospitalization was 16 days (1–72). Causes of death included progression of the oncohematologic disease (29%), sepsis, and solid tumor progression (4 patients, 24% each). Only one patient died for severe hemolytic disease. Mortality was significantly associated with sepsis (41% septic vs 11% not, *p* = 0.005) and need of platelets transfusions (59% transfusion dependent vs 15% not, *p* < 0.001). Further univariate and multivariate analyses did not highlight significant associations. Subjects’ engagement for outpatient follow-up was significantly more frequent in hematologic patients than in non-hematologic ones (*p* ≤ 0.0001).

## Discussion

Here we describe a large series of inpatients evaluated due to peripheral cytopenias admitted to non-hematological wards and show that they are mainly elderly (two-thirds > 60 years) and carry a burden of comorbidities (87% of cases) mainly involving chronic kidney and liver disease. Cytopenias were often severe, one-third of patients required transfusions, and 11% died.

After a median hospital stay of 15 days, only one-third of subjects received a discharge hematological diagnosis, equally distributed between onco- and non-oncological ones. Hematological patients showed more frequently neutropenia and thrombocytopenia as compared to non-hematological ones, whilst anemia and hemolytic markers alteration did not display significant association.

Although mortality was not significantly associated with a hematological diagnosis, up to one-third of subjects died during progression of oncohematologic disease and one due to fatal autoimmune hemolytic anemia. This data appears remarkable considering the short-term observation, highlighting the likely impact of cytopenias on survival [14, 15].

**Fig. 2:**
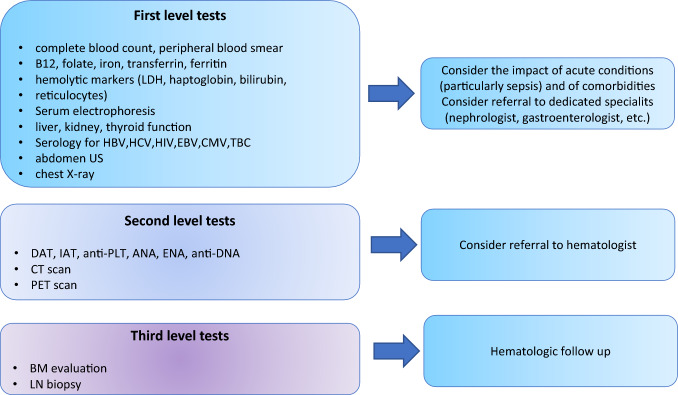
Suggested work up of cytopenias in internal medicine

Figure 2 depicts a proposed work up for cytopenias in the internal medicine setting, including first-level tests (CBC, nutrients, and organ functions), second-level (immune-hematologic and imaging), and third-level ones (BM evaluation). In our series, only one-third of thousands of tests performed were informative on the final discharge diagnosis. If “first-level” tests, mainly aimed at excluding secondary cytopenias, are generally not expensive, widely available, and autonomously ordered by the internal medicine specialist, others may be considered at a second time. These include immunohematologic tests, whose positivity was high, ranging from 4% for anti-phospholipid antibodies to 56% for anti-PLT antibodies, as in other series [16, 17]. Only DAT appeared highly sensitive and specific for AIHA diagnosis and is, therefore, recommended in case of anemia with altered hemolytic markers [18–20]. Contrarily, anti-PLT autoantibodies test confirmed to be less specific and is not routinely advised [21, 22]. Second-level imaging (CT and PET scans) appears valuable in the evaluation of cytopenic patients [23], although the main finding was the presence of infectious foci, and splenomegaly and lymph node enlargement were equally distributed among oncologic and benign diagnoses. Hematologists indicated BM aspirate and trephine in only 1/3 of cases, and this led to hematological diagnosis in about 60% of patients with persistent cytopenia of unknown cause. Notably, the most frequent finding in this setting was myelodysplasia that may be difficult to quantify, may be partly age-related [24–28], or associated with nutrients deficiencies that should be corrected before performing the test. Since most patients were elderly and with comorbidities we could not find specific factors associated with uninformative BM evaluation. In general, BM evaluation should be pursued in those patients, where it might significantly impact the clinical management and after all first- and second-level tests have been already performed. Study limitations include the retrospective nature of the study and the limited number of patients, although the detailed clinical data collected allowed to depict a real-world picture of a cytopenic internal medicine inpatient population.

In conclusion, we confirm that secondary cytopenias are more frequent in the internal medicine setting (2/3 of patients) and deserve proper workup before second/third-level hematologic tests [29]. The burden of poorly informative investigations remains an open issue depending on cytopenia severity, clinician’ expertise, as well as on availability and costs of the various tests.

### Supplementary Information

Below is the link to the electronic supplementary material.

Supplementary file1 (DOCX 96 KB)

## Data Availability

All data are available within the manuscript and further may be available upon reasonable request to the corresponding author.

## References

[1] Guralnik JM, Eisenstaedt RS, Ferrucci L, Klein HG, Woodman RC (2004). Prevalence of anemia in persons 65 years and older in the United States: evidence for a high rate of unexplained anemia. Blood.

[2] Gaskell H, Derry S, Andrew Moore R, McQuay HJ (2008). Prevalence of anaemia in older persons: systematic review. BMC Geriatr.

[3] Tettamanti M, Lucca U, Gandini F (2010). Prevalence, incidence and types of mild anemia in the elderly: the “Health and Anemia” population-based study. Haematologica.

[4] World Health Organization. Nutritional anaemias. Report of a WHO scientific group. World Health Organ Tech Rep Ser. 1968; **405**: 5–37. 4975372

[5] Pascutti MF, Erkelens MN, Nolte MA (2016). Impact of viral infections on hematopoiesis: from beneficial to detrimental effects on bone marrow output. Front Immunol.

[6] Stauder R, Valent P, Theurl I (2018). Anemia at older age: etiologies, clinical implications, and management. Blood.

[7] Weinzierl EP, Arber DA (2013). Bone marrow evaluation in new-onset pancytopenia. Hum Pathol.

[8] https://www.who.int/publications/i/item/WHO-NMH-NHD-MNM-11.1

[9] https://www.thejh.org/index.php/jh/article/view/28

[10] Aster RH (2005). Drug-induced immune cytopenias. Toxicology.

[11] Andrès E, Villalba NL, Zulfiqar AA, Serraj K, Mourot-Cottet R, Gottenberg AJ (2019). State of art of idiosyncratic drug-Induced neutropenia or agranulocytosis, with a focus on biotherapies. J Clin Med.

[12] Shander A, Javidroozi M, Ashton ME (2011). Drug-induced anemia and other red cell disorders: a guide in the age of polypharmacy. Curr Clin Pharmacol.

[13] https://publications.iarc.fr/Book-And-Report-Series/Who-Classification-Of-Tumours/WHO-Classification-Of-Tumours-Of-Haematopoietic-And-Lymphoid-Tissues-2017

[14] Denny SD, Kuchibhatla MN, Cohen HJ (2006). Impact of anemia on mortality, cognition, and function in community-dwelling elderly. Am J Med.

[15] den Elzen WP, Willems JM, Westendorp RG, de Craen AJ, Assendelft WJ, Gussekloo J (2009). Effect of anemia and comorbidity on functional status and mortality in old age: results from the Leiden 85-plus Study. CMAJ.

[16] Boren E, Gershwin ME (2004). Inflamm-aging: autoimmunity, and the immune-risk phenotype. Autoimmun Rev.

[17] Ray D, Yung R (2018). Immune senescence, epigenetics and autoimmunity. Clin Immunol.

[18] Jäger U, Barcellini W, Broome CM (2020). Diagnosis and treatment of autoimmune hemolytic anemia in adults: recommendations from the First International Consensus Meeting. Blood Rev.

[19] Fattizzo B, Barcellini W (2022). Autoimmune hemolytic anemia: causes and consequences. Expert Rev Clin Immunol.

[20] Fattizzo B, Giannotta JA, Serpenti F, Barcellini W (2020). Difficult cases of autoimmune hemolytic anemia: a challenge for the internal medicine specialist. J Clin Med.

[21] Brighton TA, Evans S, Castaldi PA, Chesterman CN, Chong BH (1996). Prospective evaluation of the clinical usefulness of an antigen-specific assay (MAIPA) in idiopathic thrombocytopenic purpura and other immune thrombocytopenias. Blood.

[22] McMillan R, Wang L, Tani P (2003). Prospective evaluation of the immunobead assay for the diagnosis of adult chronic immune thrombocytopenic purpura (ITP). J Thromb Haemost.

[23] Hughes NM, Jacene HA (2021). PET Imaging for hematologic malignancies. Radiol Clin North Am.

[24] Heibl S, Stauder R, Pfeilstöcker M (2021). Is myelodysplasia a consequence of normal aging?. Curr Oncol Rep.

[25] Valent P, Bain BJ, Bennett JM (2012). Idiopathic cytopenia of undetermined significance (ICUS) and idiopathic dysplasia of uncertain significance (IDUS), and their distinction from low risk MDS. Leuk Res.

[26] Culleton BF, Manns BJ, Zhang J, Tonelli M, Klarenbach S, Hemmelgarn BR (2006). Impact of anemia on hospitalization and mortality in older adults. Blood.

[27] Zhao J, Ghimire A, Liesveld J (2021). Marrow failure and aging: the role of “Inflammaging”. Best Pract Res Clin Haematol.

[28] Duarte FB, Barbosa MC, Jesus Dos Santos TE (2018). Bone marrow fibrosis at diagnosis is associated with TP53 overexpression and adverse prognosis in low-risk myelodysplastic syndrome. Br J Haematol.

[29] Zaninetti C, Klersy C, Scavariello C, Bastia R, Balduini CL, Invernizzi R (2018). Prevalence of anemia in hospitalized internal medicine patients: correlations with comorbidities and length of hospital stay. Eur J Intern Med.

