# Supplementary material for: Deciphering cytopenias in internal medicine: a single-center observational study
Source: Intern Emerg Med. 2024 Jan 25;19(3):661–8. doi: 10.1007/s11739-023-03517-z (PMC11039542; doi:10.1007/s11739-023-03517-z)
Supplement: Supplementary file 1 — Supplementary file1 (DOCX 96 KB) [file 11739_2023_3517_MOESM1_ESM.docx]

**Supplementary tables**

| ***Supplementary table 1*. Comorbidities** | All | Non-hematologic | Hematologic |
| --- | --- | --- | --- |
|  | N=151 | N=98 | N=53 |
| **Cardiovascular disease** | 89 (58.9%) | 51 (52%) | 38 (71.7%) |
| Congestive heart failure | 8 (8.9%) | 5 (9.8%) | 3 (7.9%) |
| Arrhythmias | 30 (33.7%) | 16 (31.4%) | 14 (36.8%) |
| Ischemic cardiomyopathy | 19 (21.3%) | 10 (19.6%) | 9 (23.7%) |
| Other cardiomyopathies | 6 (6.7%) | 2 (3.9%) | 4 (10.5%) |
| Arterial hypertension | 66 (74.2%) | 34 (66.7%) | 32 (84.2%) |
| Peripheral vasculopathy | 5 (5.6%) | 3 (5.9%) | 2 (5.3%) |
| Valvulopathy | 11 (12.4%) | 8 (15.7%) | 3 (7.9%) |
| **Kidney disease** | 36 (23.8%) | 24 (24.5%) | 12 (22.6%) |
| **Hepatic disease** | 63 (41.7%) | 49 (50%) |  |
| Acute hepatitis | 24 (40.7%) | 17 (34.7%) | 7 (13.2%) |
| Chronic hepatopathy | 39 (66.1%) | 32 (65.3%) | 8 (15.1%) |
| Cirrhosis | 24 (61.5%) | 19 (59.4%) | 5 (62.5%) |
| Hepatocellular carcinoma | 4 (10.3%) | 4 (12.5%) | 0 |
| **Intravascular devices** | 5 (3.3%) | 3 (3.1%) | 2 (3.8%) |
| **Autoimmune disease** | 30 (19.9%) | 18 (18.4%) | 12 (22.6%) |
| **Diabetes mellitus** | 37 (24.5%) | 25 (25.5%) | 12 (22.6%) |
| **Thyroid disease** | 33 (21.9%) | 1 (1.0%) | 15 (28.3%) |
| **Sepsis** | 23 (15.2%) | 17 (17.3%) | 6 (11.3%) |
| **Thrombosis** | 14 (9.3%) | 8 (8.2%) | 6 (11.3%) |

| **Supplementary table 2. Bone marrow evaluation** | All  N=46 | Non-hematologic  N=14 | Hematologic  N=32 |
| --- | --- | --- | --- |
| **Cellularity** | 50 (10-95) | 35 (10-90) | 50 (15-95) |
| **Diserythropoiesis** |  |  |  |
| Present | 37 (80.4%) | 11 (78.6%) | 26 (81.3%) |
| Absent | 9 (19.6%) | 3 (21.4%) | 6 (18.7%) |
| **Dismegakaryocytopoiesis** |  |  |  |
| Present | 8 (17.4%) | 1 (7.1%) | 7 (21.9%) |
| Absent | 38 (82.6%) | 13 (92.9%) | 25 (78.1%) |
| **Disgranulocytopiesis** |  |  |  |
| Present | 6 (13%) | 2 (14.3%) | 4 (12.5%) |
| Absent | 40 (87%) | 12 (85.7%) | 28 (87.5%) |
| **Fibrosis** |  |  |  |
| MF-1 | 4 (8.7%) | 1 (7.1%) | 3 (9.4%) |
| MF-2 | 1 (2.2%) | 0 | 1 (3.1%) |
| MF-3 | 0 | 0 | 0 |
| Absent | 41 (89.1%) | 13 (92.9%) | 28 (87.5%) |
| **Blasts** | 2 (0-35) | 1 (0-3) | 2 (0-35) |
| **Infiltrate** |  |  |  |
| B lymphocytes | 1 (2.2%) | 0 | 1 (3.1%) |
| T lymphocytes | 7 (15.2%) | 1 (7.1%) | 6 (18.8%) |
| Mixed cellular | 26 (56.5%) | 8 (57.2%) | 18 (56.3%) |
| Clonal infiltrate | 4 (8.7%) | 0 | 4 (12.5%) |
| Absent | 12 (26.1%) | 5 (35.7%) | 7 (21.8%) |
| **Percentage of lymphoid infiltrate** | 5 (0-25) | 4 (0-5) | 5 (0-25) |

| **Supplementary table 3. Causes of death and demographics** | Dead  N=17 (11.3%) | Survivors  N=134 | *p* |
| --- | --- | --- | --- |
| Oncohematologic disease | 5 (29.4%) | 22 (16.4%) | 0.311 |
| Immunohematologic disease | 1 (5.9%) | 25 (18.7%) | 0.308 |
| Infection | 4 (23.5%) | 49 (36.7%) | 0.419 |
| Solid tumor | 4 (23.5%) | 19 (14.2%) | 0.472 |
| Age | 70 (19-94) | 71(15-96) | 0.993 |
| Males | 8 (47.1%) | 76 (56.7%) | 0.605 |
| Females | 9 (52.9%) | 58 (43.3%) | - |
| Duration of hospitalization | 16 (1-72) | 15 (2-166) | 0.672 |

**Supplementary figures**

**Supplementary figure 1. Patient disposition.**
